# Sudden Cardiac Death in a Patient with Thrombotic Thrombocytopenic Purpura: A Case Report

**DOI:** 10.3390/hematolrep14020027

**Published:** 2022-06-02

**Authors:** Kikuaki Yoshida, Shogo Murata, Masaya Morimoto, Toshiki Mushino, Ken Tanaka, Yusuke Yamashita, Hiroki Hosoi, Akinori Nishikawa, Shinobu Tamura, Kinta Hatakeyama, Masanori Matsumoto, Takashi Sonoki

**Affiliations:** 1 Department of Hematology/Oncology, Wakayama Medical University, Wakayama 6418509, Japan; kyoshida@wakayama-med.ac.jp (K.Y.); masamor@wakayama-med.ac.jp (M.M.); mushino@wakayama-med.ac.jp (T.M.); ken-t@wakayama-med.ac.jp (K.T.); yyyamash@wakayama-med.ac.jp (Y.Y.); h-hosoi@wakayama-med.ac.jp (H.H.); nishikaw@wakayama-med.ac.jp (A.N.); stamura@wakayama-med.ac.jp (S.T.); sonoki@wakayama-med.ac.jp (T.S.); 2 Department of Pathology, National Cerebral and Cardiovascular Center, Osaka 5648565, Japan; kpathol@ncvc.go.jp; 3 Department of Blood Transfusion Medicine, Nara Medical University, Nara 6348521, Japan; mmatsumo@naramed-u.ac.jp

**Keywords:** thrombotic thrombocytopenic purpura, inhibitor boosting, cardiac death

## Abstract

A 49-year-old female was admitted to our hospital with malaise and gross hematuria. As ADAMTS13 (a disintegrin-like and metalloproteinase with thrombospondin type 1 motifs 13) activity was absent and the ADAMTS13 inhibitor was detected, she was diagnosed with acquired thrombotic thrombocytopenic purpura (TTP). In addition to plasma exchange and corticosteroid therapy, she received rituximab therapy for inhibitor boosting but died suddenly of a cardiac arrest on day 9. The postmortem revealed microvascular platelet thrombi in multiple organs. In this case, the deterioration of the patient’s clinical status was considered to have been caused by inhibitor boosting-induced systemic microvascular occlusion. In particular, her sudden death may have been due to cardiovascular microthrombosis. Since inhibitor boosting can cause TTP patients to deteriorate rapidly, it is crucial to manage TTP patients who undergo inhibitor boosting appropriately. The monitoring of cardiac complications in TTP patients may also be essential, especially in the acute phase.

## 1. Introduction

Thrombotic thrombocytopenic purpura (TTP) can cause life-threatening thrombotic microangiopathy by inducing severe deficiency in ADAMTS13 (a disintegrin-like and metalloproteinase with thrombospondin type 1 motifs 13) activity. ADAMTS13 physiologically cleaves multimers of von Willebrand factor (VWF), a glycoprotein that is necessary for normal hemostasis [1,2]. TTP is characterized by platelet aggregation and extensive microvascular thrombosis associated with a deficiency of ADAMTS13 activity. It is classified into congenital and acquired forms, both of which exhibit ADAMTS13 activity levels of <10%, and acquired TTP also involves the production of anti-ADAMTS13 autoantibodies (ADAMTS13 inhibitor) [3]. Patients with acquired TTP are treated with plasma exchange (PE) using fresh frozen plasma (FFP) to replenish ADAMTS13 and remove ADAMTS13 inhibitor, which markedly reduces the risk of mortality [4]. Corticosteroid therapy may be given in combination with PE to suppress autoantibody production. Recently, the monoclonal anti-CD-20 antibody rituximab (RTX) has been shown to be effective against refractory or relapsed TTP [5]. Herein, we report a case of refractory TTP involving sudden death due to inhibitor boosting. The patient was treated with PE, corticosteroids, and RTX, but the disease could not be controlled, and the patient died. A postmortem study revealed microthrombi in multiple organs. It is plausible that small vessel occlusion in multiple organs, and, more directly, cardiac pump failure, contributed to the patient’s death. Clinicians should be aware that TTP can rapidly worsen, even when standard therapies such as PE are administered promptly. Furthermore, we consider that the patient’s cardiac status is of critical importance in the acute phase of TTP.

## 2. Case Report

A 49-year-old female developed malaise, nausea, and gross hematuria over several days and visited a nearby hospital. A laboratory examination showed marked thrombocytopenia (platelet count: 10 × 10^9^/L). She was immediately transferred to our hospital with a suspected hematological disorder. She had been suffering from primary Sjögren’s syndrome with mouth dryness for 2 years. On admission, she did not have any disturbances of consciousness, her body temperature was 37.3 °C, and her other vital signs were normal. The laboratory data obtained upon admission to our hospital are shown in Table 1. They were indicative of severe thrombocytopenia, hemolytic anemia, and renal dysfunction. Blood cultures, other cultures, and computed tomography (CT) without contrast medium revealed no evidence of infectious disease or malignancy. The patient’s ADAMTS13 activity and ADAMTS13 inhibitor levels were <0.5% (reference value 40–130%, enzyme-linked immunosorbent assay (ELISA)) and 5.1 Bethesda units (BU)/mL (reference value < 0.5 BU/mL, Bethesda assay), respectively. ADAMTS13 antigen was not measured. Based on these findings, we diagnosed her with acquired TTP.

**Figure 1:**
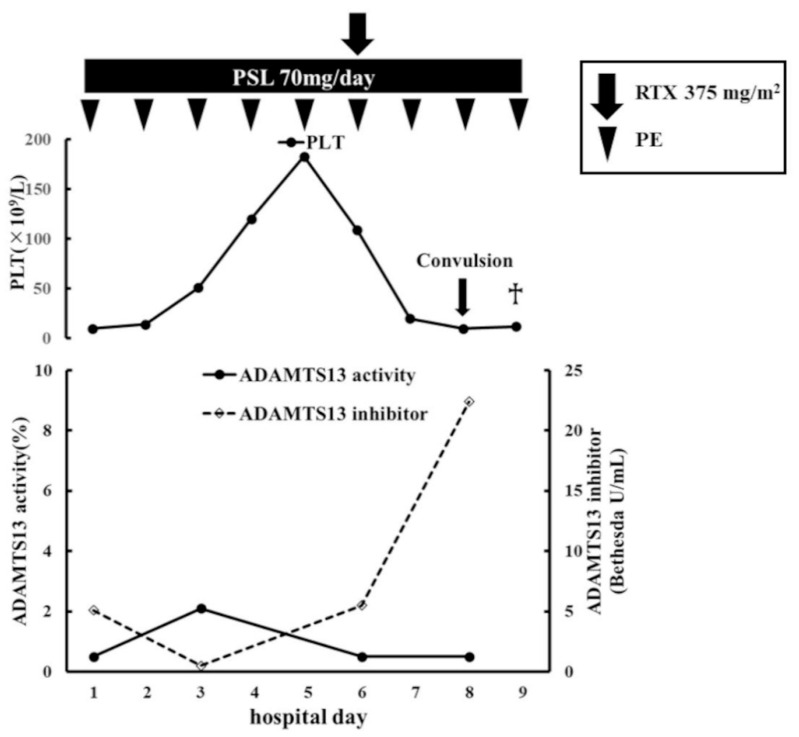
Clinical course of the current case after admission. The daily PE and PSL were continued and RTX was added on the 6th day. The patient’s platelet count normalized on the 5th day, but it began to decrease again on the 6th day and did not recover. Her ADAMTS13 activity only slightly recovered (to 2.1%) and her ADAMTS13 inhibitor level initially decreased, but significantly increased again after 5 days. The patient developed systemic tonic convulsions and entered the ICU on the 8th day. On the 9th day, she died of a cardiac arrest due to a sudden drop in blood pressure. PE: plasma exchange therapy; PSL: prednisolone; PLT: platelets; RTX: rituximab; BU: Bethesda units; ICU: intensive care unit.

From the day of admission, the patient was initially treated with PE and corticosteroid therapy (70 mg/day intravenous prednisolone (PSL)) for suspected TTP. By the 5th hospital day, her platelet count had increased to 183 × 10^9^/L and her lactate dehydrogenase (LDH) level had returned to a normal range. Until then, she had exhibited an uneventful course. However, her platelet count decreased to 109 × 10^9^/L on the 6th hospital day, which was suggestive of refractory TTP with inhibitor boosting. Then, her ADAMTS13 inhibitor levels increased again. She was administered 375 mg/m^2^ of RTX immediately, but her platelet count continued to decrease. On the 8th day, she presented with a headache and dysarthria. Brain CT and magnetic resonance imaging without contrast medium did not reveal any signs of a stroke. She developed systemic tonic convulsions on the same day, after which she entered the intensive care unit (ICU) due to a disturbance of consciousness. At that time, her consciousness level was E4V4M6 according to the Glasgow Coma Scale (GCS). Serological analysis showed that her ADAMTS13 inhibitor titer had risen to 22.4 BU/mL. She experienced convulsions once more, and then her consciousness level deteriorated (GCS: E2V2M5). She was intubated on the 9th day due to the risk of airway obstruction. She was found to have progressive lactic acidosis of unknown cause, suggesting that her physical state was worsening rapidly. Shortly thereafter, she was unable to maintain adequate blood pressure, leading to cardiac arrest. The electrocardiogram showed pulseless electrical activity, and resuscitation was performed, but the patient did not respond. Finally, she died a few hours after she was intubated due to low blood pressure (Figure 1).

**Figure 2:**
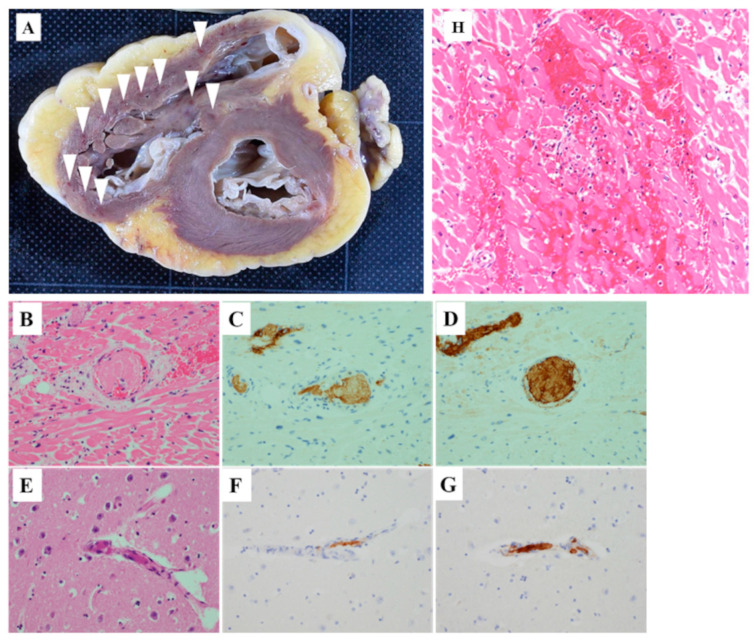
(**A**) Macroscopic findings of the heart. Many petechial hemorrhagic lesions were observed in the right ventricle wall (arrowhead). (**B**–**G**) Postmortem pathological findings of the heart and brain. Hematoxylin-eosin staining of the heart showed microthrombi in the small blood vessels with focal hemorrhaging in the surrounding myocardium (**B**). The cells were slightly fibrin-positive (**C**) and markedly VWF-positive (**D**) (magnification: 200×). Similarly, hematoxylin-eosin staining of the brain also revealed occlusive thrombi (**E**). The thrombi were slightly fibrin-positive (**F**) and markedly VWF-positive (**G**) (magnification: 200×). Bleeding and necrosis of cardiomyocytes and inflammatory cell infiltration (**H**) (magnification: 200×).

A postmortem examination revealed microscopic microthrombi in multiple organs, including the heart, brain, and kidneys, in addition to mucocutaneous petechiae throughout the body. Macroscopically, the heart showed many petechial hemorrhagic lesions in the myocardial walls, but no obvious myocardial necrosis (Figure 2A), and only a small amount of pericardial effusion was present. In the brain, there was no apparent hemorrhaging or infarction. No obvious necrotic lesions were found in the intestinal tract or other parts of the body, and there were no neoplastic lesions that could have caused TTP. Microscopically, there were numerous microvascular thrombi in the heart. In the brain, there were also many microthrombi in the cerebrum, pons, and medulla oblongata. Similar microthrombi were found in various organs, including the liver and kidneys. The microthrombi were weakly stained for fibrin but contained a large quantity of VWF, which was consistent with VWF-rich platelet thrombi (Figure 2B–G). The worsening of the patient’s clinical status due to microvascular occlusion throughout the body, and, more directly, cardiac pump failure was considered to have contributed to her sudden death.

## 3. Discussion

We consider that the course of our case provides clinically instructive findings from several perspectives. For example, the patient suddenly died after her condition deteriorated rapidly due to inhibitor boosting. In addition, it was pathologically proven that microthrombosis caused by TTP directly contributed to her death.

First, TTP can have a rapid course due to inhibitor boosting. According to the most recent definition, a clinical response is defined as a sustained platelet count of ≥150 × 10^9^/L, an LDH level of <1.5 times the upper limit of normal, and no clinical evidence of a new or progressive ischemic organ injury [6]. Based on the above definition, our patient did not exhibit a clinical response. We considered inhibitor boosting to be one of the reasons for the inadequate treatment response. A recent study described inhibitor boosting as a reduction followed by a rise in the ADAMTS13 inhibitor level after PE therapy, which may occur due to the exogenic ADAMTS13 present in FFP being recognized as an antigen during PE [7]. Our patient, who was initially treated with PE and corticosteroids, experienced a rapid deterioration in her TTP. Her ADAMTS13 inhibitor level decreased from 5.1 BU/mL to <0.5 BU/mL but then increased to 5.5 BU/mL on the 6th day. This course of events could be explained by inhibitor boosting. Furthermore, her ADAMTS13 inhibitor level subsequently increased markedly to 22.4 BU/mL despite continued treatment and the addition of RTX. It was reported that the early use of RTX was effective in some cases of inhibitor boosting [8,9]. In the current case, RTX was administered on the 6th day when the patient’s platelet count started to decrease. RTX suppresses the production of ADAMTS13 inhibitor by reducing the number of B lymphocytes, but this may take several days [10]. Although RTX did not improve disease progression in this case, it is unclear whether it was maximally effective because the patient died a few days after its administration. Moreover, it should be emphasized that the patient’s death was not caused by a delay in diagnosis or treatment. In this case, PE was performed immediately after the patient arrived at our hospital, and RTX was promptly administered under suspicion of inhibitor boosting. Despite these treatments, the patient died soon after. It is important to bear in mind that TTP can have an acute course due to inhibitor boosting.

Secondly, the cause of the patient’s death was pathologically demonstrated to be TTP-induced microthrombi. The postmortem revealed microthrombi containing large amounts of VWF which were considered to be TTP-induced platelet thrombi in multiple organs. We considered that cardiac disorders were heavily involved in the sudden death of the patient. In the heart, no macroscopic infarction or hemorrhaging was noted. However, the patient’s cardiovascular findings included widespread platelet thrombi in the small vessels and many petechial hemorrhagic lesions in the myocardial walls, suggesting that a cardiac injury due to thrombosis may have been the immediate cause of death. A pathological examination of the brain showed the widespread distribution of microthrombi in the cerebrum, cerebellum, pons, and medulla oblongata, but no obvious infarction foci. The patient had no history of epilepsy or other brain disorders, suggesting that she had developed convulsions due to diffuse TTP-induced cerebral thrombosis. The patient experienced convulsions on two occasions. Neither of these convulsions were persistent—i.e., they disappeared within about one minute. Microthrombi in the brain may have contributed to the onset of the convulsions but were not considered to be a cause of the patient’s death. There was no other evidence of a direct cause of death, such as extensive necrosis. In conclusion, systemic thrombosis was considered to be a plausible cause of the patient’s worsening clinical course. In addition, it is very likely that cardiogenic shock due to cardiovascular microthrombi contributed to her sudden death.

Cardiovascular manifestations that are masked by clinical deterioration are often overlooked. Postmortem studies of TTP patients have found intramyocardial microthrombi in the majority of cases [11]. Therefore, it is conceivable that most TTP patients are at risk of myocardial damage from microthrombosis. There has been a case report of sudden cardiac death in TTP patients due to heart failure [12]. Optimal management strategies for cardiovascular microthrombi have not yet been determined. Based on our experience, we suggest that when TTP is diagnosed the patient should be carefully evaluated for cardiac complications.

Our patient had a history of primary Sjögren’s syndrome (SS). In addition, there were no other exact causes of autoimmune TTP, such as malignancies, infections, pregnancies, or other autoimmune diseases. In this regard, it is conceivable that the cause of TTP in our case was related to primary SS. To the best of our knowledge, 19 cases of TTP with SS have been reported in the past 55 years, and only 7 of them were diagnosed with SS prior to TTP, as in our case [13,14]. In an analysis of 261 cases of acquired TTP, 56 patients were reported to have developed autoimmune diseases, including systemic lupus erythematosus (SLE) and SS [15]. We believe that more attention should be paid to the history of SS in patients with TTP.

We encountered a case of TTP involving sudden death due to microthrombosis. Despite the use of RTX and PE, the mortality rate of patients with acquired TTP remains between 10 and 20% [16]. The mainstay of the current treatment strategy for TTP is to remove ADAMTS13 inhibitor using PE and prevent ADAMTS13 inhibitor production using immunosuppressive therapy. However, since these therapies act on the underlying autoimmune process, they may take some time to produce a response. We consider that in cases of TTP involving acute deterioration, existing therapies may not be sufficient to reduce the risk of mortality because the degree of organ damage caused by thrombosis can be unpredictable. Recently, the efficacy of newer therapies has been examined. As an example, caplacizumab, which blocks VWF-mediated microthrombi formation, may prevent short-term peripheral vascular ischemia more quickly than conventional treatment alone [17]. However, as of now, this drug is not generally administered, as it could not be covered by Japanese health insurance. There is a need for further studies to identify strategies for improving the prognosis of TTP.

Our case report has some limitations. Unfortunately, we could not investigate whether the patient had clinical cardiac symptoms, such as chest pain, just before her death. We also could not perform clinical cardiac evaluations, including the use of cardiac biomarkers, electrocardiograms, and echocardiography. Cardiological examinations including echocardiography might have provided the findings to identify her cardiac complications earlier.

## 4. Conclusions

In conclusion, inhibitor boosting in TTP can lead to hyperacute exacerbation due to microvascular thrombosis in multiple organs. Cardiac evaluations of TTP patients might need to be carried out more carefully, especially in the acute phase.

## Figures and Tables

**Table 1:** Laboratory data obtained on admission to our hospital.

Complete Blood Count			Blood Chemistry		
WBC	10.3	×10^9^/L	AST	98	IU/L
RBC	3.21	×10^12^/L	ALT	36	IU/L
Hb	89	g/L	LD	2966	IU/L
Ht	25.4	%	T-Bil	2.1	mg/dL
MCV	79.1	fL	D-Bil	0.3	mg/dL
Ret	164	×10^9^/L	Creatinine	2.17	mg/dL
Plt	19	×10^9^/L	BUN	39.9	mg/dL
Coagulation System			CRP	6.75	mg/dL
APTT	29.9	Sec	Haptoglobin	3	ng/mL
PT time	12.3	Sec	Schistocytes	15	%
Activity	82.2	%	cTnI (0–26.2)	55.4	pg/mL
PT-INR	1.11				
Fib	5.24	g/L	ADAMTS13 activity (40–130)	<0.5	%
FDP	28.6	mg/L	ADAMTS13 inhibitor (<0.5)	5.1	BU/mL

Ret: reticulocytes; PT: prothrombin time; Activity: prothrombin activity; AST: aspartate transaminase; ALT: alanine transaminase; LD: lactate dehydrogenase; T-Bil: total bilirubin; D-Bil: direct bilirubin; BUN: blood urea nitrogen; cTnI: cardiac troponin I; ADAMTS13: a disintegrin-like and metalloproteinase with thrombospondin type 1 motifs 13; BU: Bethesda units.

## Data Availability

The data used and analyzed during the current study are available from the corresponding author on reasonable request.
